# Editorial: Kidney replacement therapy advances in children

**DOI:** 10.3389/fped.2022.970884

**Published:** 2022-07-19

**Authors:** Rupesh Raina, Sidharth Kumar Sethi, Nikhil Nair, H. K. Yap

**Affiliations:** ^1^Pediatric Nephrology, Akron Children's Hospital, Cleveland, OH, United States; ^2^Pediatric Nephrology, Kidney Institute, Medanta, The Medicity Hospital, Gurgaon, Haryana, India; ^3^Akron Nephrology Associates, Cleveland Clinic Akron General Medical Center, Akron, OH, United States; ^4^Department of Pediatrics, Yong Loo Lin School of Medicine, National University of Singapore, Singapore, Singapore

**Keywords:** acute kidney injury, critical care pediatric nephrology, critical care, kidney replacement therapy, dialysis, pediatric dialysis, infant dialysis, continuous kidney replacement therapy

In cases of severe Pediatric Acute Kidney Injury (AKI), the initiation of dialysis techniques such as peritoneal dialysis (PD), hemodialysis (HD), continuous kidney replacement therapy (CKRT), and newly available technologies made especially for children, such as, sustained low-efficiency dialysis (SLED)/SLED-f, NIDUS, CARPEDIEM, and Aquadex may prove to be more efficacious and help to preserve renal function longer than non-dialytic techniques. AKI incidence in the pediatric population has been increasing significantly. Over the previous years, the advancement in development and utilization of KRT allows for protection of pulmonary and cardiac systems as well. Changes and updates to new biocompatible solutions and updates to flow settings in ultrafiltration have allowed for increased fluid removal with decreased adverse effects (1). CKRT modalities allow increased and continuous clearance of waste products and cytokines until the patient returns to homeostasis. These modalities have been shown to be especially effective in the case of fluid overload and preventing adverse outcomes and further renal decline. Advancements in biofilters can more specifically remove cytokines and endotoxins (2). Hybrid techniques such as sustained low-efficiency daily hemodiafiltration (SLED-f) allow for clearance of small as well as larger solutes and less need for anti-coagulants in critical children. In adult cohorts, new dialyzers that utilize convection and diffusion allow for the production of ultrapure filtrates to further increase clearance rates. These modalities are starting to be investigated for efficacy within the pediatric population. These advances and further adaptation of successful innovations in the adult population for the pediatric population require more studies and analysis. In the end, the choice of KRT techniques should be based on the healthcare team's resources and expertise.

The Research Topic “*Kidney Replacement Therapy Advances in Children*” includes novel and original contributions in the field discussing utilization of updates to KRT techniques through workflow and dialysis settings to reduce the risk of AKI development. Many of the works are conducted by the members of the PCCRT-ICONIC group who have been researching and advancing knowledge on Renal Replacement therapies in the pediatric population. A history of the group and the guidelines previously published are shown in Figure 1. This current Research Topic highlights a mix of retrospective analysis, prospective studies and systematic reviews to examine the efficacy of current standards of care to highlight new ways forward for treatment and research. In this Research Topic issue of *Frontiers*, we bring together a special collection of 6 articles contributing sound evidence for the key concepts outlined.

**Figure 1:**
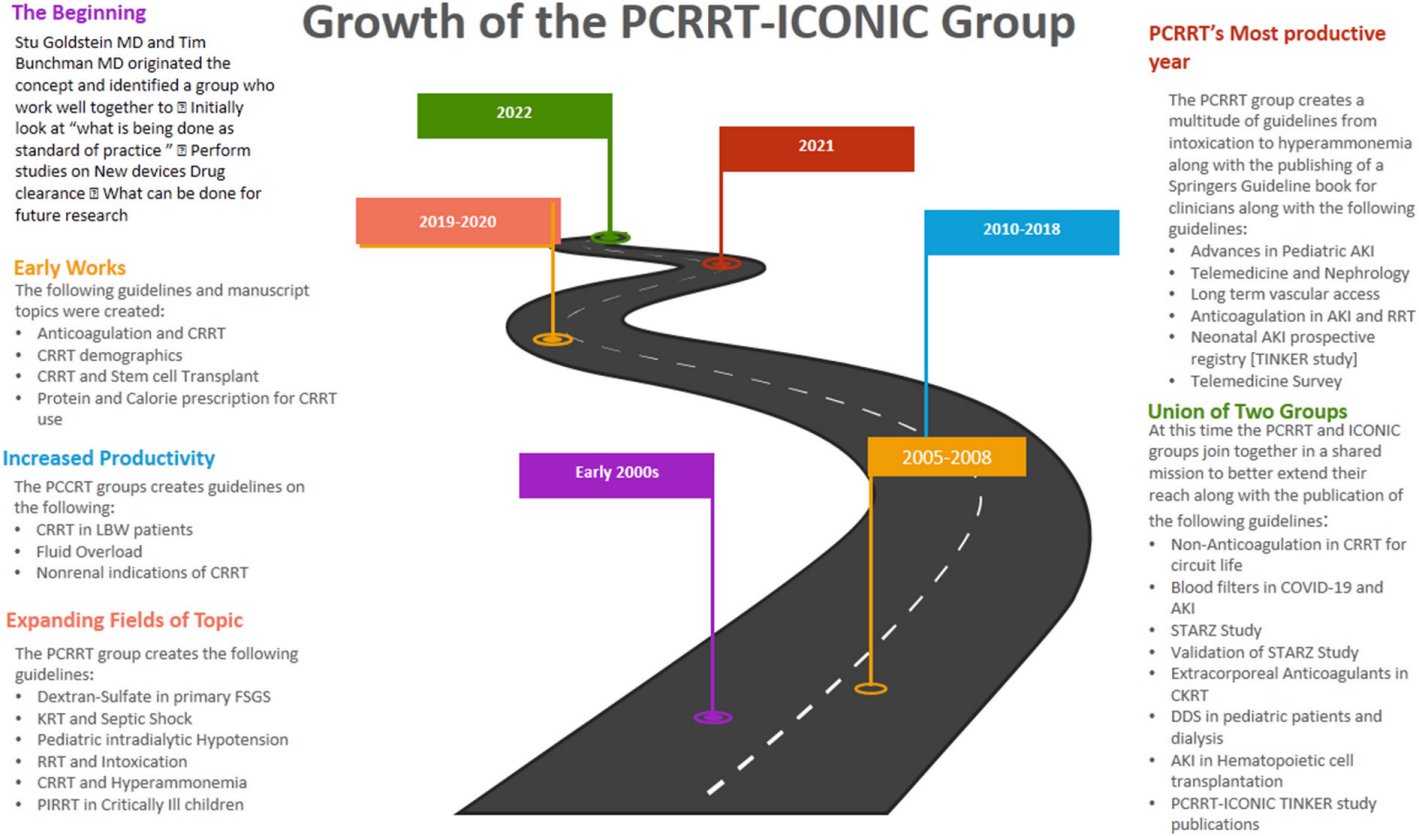
A roadmap of the formation of the PCCRT-ICONIC group and previous topic areas covered in guidelines and manuscripts written by group members. AKI, Acute kidney injury; CRRT, Continuous renal replacement therapy; DDS, Dialysis disequilibrium syndrome; LBW, Low-Birth weight; FSGS, Focal segmental glomerulosclerosis; PIRRT, Prolonged intermittent renal replacement therapy; STARZ, Sethi, Tibrewal, Agarwal, Raina, and Wazir; TINKER, The Indian PCCRT-ICONIC neonatal kidney education registry.

This issue opens with “*Re-evaluating Renal Angina Index: An Authentic, Evidence-Based Instrument for Acute Kidney Injury Assessment: Critical Appraisal*” by, Raina, Sethi, Mawby, et al. Clinically the diagnosis of AKI has been done through an acute increase in Serum creatinine along with the presence of oliguria, though it is neither specific nor sensitive. The renal angina index (RAI) has been utilized and explored used to predict the risk of AKI development through addition of more clinical context and AKI risk factors. Raina, Sethi, Mawby, et al. and the PCCRT-ICONIC group do a sweeping review on the use of RAI in the literature and its functionality as a predictor of AKI with updates on its sensitivity and specificity. Next Fang et al. investigates the “*Clinical Features and Risk Factors of Fungal Peritonitis in Children on Peritoneal Dialysis*.” In their manuscript, they analyze the clinical manifestation, etiology, prognosis, and risk factors for fungal peritonitis for pediatric patients on PD. An extensive retrospective analysis was conducted highlighting the rare nature of the pathology and its relation to technique failure.

Agrawal et al. highlight the “*Incidence, Risk Factors, and Outcomes of Neonatal Acute Kidney Injury: Protocol of a Multicentric Prospective Cohort Study [The Indian Iconic Neonatal Kidney Educational Registry (TINKER)]*.” This manuscript dives deeper into the effects of AKI on neonate populations which has a paucity of literature and data on KRT modalities. Much of the modalities utilized are based on their role in pediatric and adolescent populations. This multicentric, national, prospective cohort study conducted by members of the TINKER [a sub-group of PCCRT-ICONIC group] enrolled an incredible 2,000 neonates with extensive clinical and demographic data taken. This data is combined to find risk factors, clinical characteristics and outcomes of AKI in this population that can be utilized by healthcare teams to recognize the early signs of AKI development in neonates. They also postulate further areas of studies and advancement in this population.

Bottari et al. discusses the “*Role of Hemoperfusion with CytoSorb Associated With Continuous Kidney Replacement Therapy on Renal Outcome in Critically Ill Children With Septic Shock*” Sepsis related AKI is a significant cause of morbidity and mortality. Inflammatory mediators represent a significant mediator in the pathophysiology of Sepsis related AKI. A retrospective analysis of CytoSorb Mediated Hemoperfusion was studied by the authors to see their effect on removing cytokines and medium sized molecules that stipulate cytokine storms. The authors found promising results from the use of this technique to help preemptively stop the development of sepsis mediated renal failure.

Raina, Sethi, Filler, et al. then highlights the “*PCRRT Expert Committee ICONIC Position Paper on Prescribing Kidney Replacement Therapy in Critically Sick Children With Acute Liver Failure*” Managing acute liver failure and acute on chronic liver failure in this population can be difficult. These patients are specifically at risk for hepatorenal syndrome and AKI which can be significant for poor prognosis. There is a paucity of literature on this pathology, however, the team of Raina, Sethi, Filler, et al. and the PCCRT-ICONIC group scour the literature to provide clinical guidance and practice points on the use of CKRT to help healthcare teams optimize clinical outcomes and to preserve renal function as long as possible. They additionally postulate areas of future research and improvement into this issue.

Pais and Wightman then end the issue by “*Addressing the Ethical Challenges of Providing Kidney Failure Care for Children: A Global Stance*.” Aside from the clinical challenges in regard to KRT use, and aspect not as highlighted is that of the psychosocial relationship between the patient and their family. Many of these patients are not able to give informed consent and rely on their patients to make important healthcare choices. Pais and Wightman give a comprehensive overview of the ethical challenges that lie with lack of equity in low and middle income nations and end with key action items and practice points for pediatric nephrologists in these positions.

In summary, this Research Topic provides original articles and reviews that may add new information on the epidemiology, pathophysiology, diagnosis and management of AKI through KRT modalities in the pediatric population. Additionally, it is our hope that these collected articles may spur additional research into these important topics.

## Author contributions

All the authors contributed equally to the draft and review of the current article and approved the submitted version.

## Conflict of interest

The authors declare that the research was conducted in the absence of any commercial or financial relationships that could be construed as a potential conflict of interest.

## Publisher's note

All claims expressed in this article are solely those of the authors and do not necessarily represent those of their affiliated organizations, or those of the publisher, the editors and the reviewers. Any product that may be evaluated in this article, or claim that may be made by its manufacturer, is not guaranteed or endorsed by the publisher.
